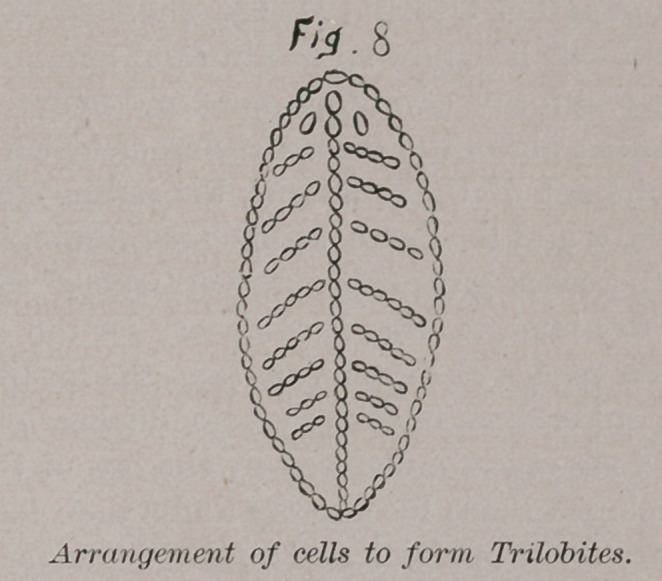# Causes of Change in Animal Forms

**Published:** 1883-01

**Authors:** Hubbard W. Mitchell

**Affiliations:** Professor of Comparative Anatomy in the Columbia Veterinary College


### ART. IV.—CAUSES OF CHANGE IN ANIMAL FORMS.

BY HUBBARD W. MITCHELL, M.D.

*Professor of Comparative Anatomy in the Columbia Veterinary College.*

IN those early geological ages when our globe was enveloped by a universal ocean it is not probable that any form of life existed beneath the surface of the sea. That long age, beginning at the very moment when our planet was thrown off by a stupendous chemical process from the central mass of incandescent gas, or sun, and continuing through inconceivable years during the slowly cooling and super-crusting of the earth, down to the time when it became cool enough to allow the waters to rest upon its surface, was called the Archœan Age, or Azoic Age, and was indeed the Beginning, or an age or epoch without life.

The first dry land that reared its head above this wide, shoreless and lifeless sea was at the Lower Silurian Age. It was a long line of low hills, commencing on the northeast at Labrador, extending southerly and westerly to the region of upper New York and the Great Lakes, then bending to the northwest and stretching away to the Arctic ocean. This bow-shaped strip of land has been named the Laurentian Hills, from the fact of its lying partly along the border of the St. Lawrence River and Gulf.

It formed a great barrier that divided a limitless expanse of waters into a northern and southern ocean. This early uplift of land has remained quiet and stable through all subsequent ages—ages that have witnessed the most tremendous convulsions of nature—the successive risings and sinkings of continental areas, the mighty upheavals of vast mountain chains, the fearful bendings, and foldings, and distortions of the earth's crust, down to our own times.

That early Silurian beach was wide-stretching and desolate, and silent. In its earliest dawn it was devoid of life. Climates did not exist, a temperature nearly uniform encompassed the earth. Deluging rains fell upon it, but the thick atmosphere, heavily laden with carbonic acid gas, was nearly quiescent.

Life under such conditions was almost impossible. Only the very lowest forms—if even these—could exist. When did life first appear on the earth, and how did it first appear?

Fossil remains of a very low order tell us that it appeared *sometime* during this lower Silurian age, sometime after the first uprising of the Laurentian Hills. But as to *how* it appeared is another and more difficult question. It is a question, however, that will force itself upon our minds, and thoughtful men earnestly strive to find an answer to it. As science pushes its remorseless and searching inquiries farther and farther, new forms of life are discovered, each simpler in construction and plan than the preceding forms until the simplest of all has been found, namely—a single cell. The line seems almost complete from, beginning almost anywhere in the animated scale, the Protozoans, the Sponges, the Algæ, and the multitude of systemless animals, composed of a few cells, down to an animal composed of one.

It is the now generally received opinion among scientific men that the cell was the first form of life that appeared on the earth, and that all subsequent forms have been modified from it. But where did the first cell come from and of what is it composed.

Chemists tell us that a single atom of oxygen gas has the power to attract to itself two atoms of hydrogen gas, and the union of these three atoms of gas form water, a substance totally different from the two impalpable gases that compose it. And if a single atom of nitrogen gas be united to five atoms of oxygen gas we have a strange substance as a result, namely—Nitric Acid, N. O .

If we take a certain number of atoms of C. H. O. and unite them we have cane sugar; by varying the number of atoms of each we can form a long list of totally dissimilar substances.

Four atoms of carbon united with five of hydrogen give us an important substance, a compound radical called Ethyl, which is thus expressed C $H^5$.

If to this compound a single atom of oxygen be added we have Ether $C^4 H^5 O$. By adding to this H. O., a single atom of each we have the well known substance alcohol—$C^4 H^6 O^2$.

The addition of nitrogen in varying proportions, to C. H. O.

in varying proportions, gives us a long array of strange and curious and interesting bodies well known to organic chemistry.

This aptitude that certain atoms have to combine with certain other atoms to produce these strange compounds is called chemical affinity. It is a subtle power, absolute in its operation, that causes simple atoms to rush together and arrange themselves in such a way as to make new and unlike substances possessing properties entirely distinct from the original elements.

Knowing then that this law of chemical affinity exists, that by its operation simple elements unite to form complex bodies with *higher powers* we are led to inquire if the *simple cell* may not have been formed by the process of some such law, and received its powers of growth and multiplication by what may be called *chemical force.*

Bearing this query in view, I will venture to offer the following theory:

A given number of atoms of C. H. N. O. combine and arrange themselves in such a way as to form a minute mass of a jelly-like substance (which has already received the name of Protoplasm.) This mass, floating on or in the water, gradually acquires the power to attract to itself simpler elements which it appropriates and uses as food. Through this power to assimilate food it acquires another, that of dividing and multiplying itself so as to form other masses.

These masses may be called cells.

Cells thus formed by the chemical union of simple elements and acquiring the power of *receiving food* and of *multiplication* and *growth*, also acquire a peculiar *vitality* enabling them to exist by themselves, of increasing in numbers, of attracting other cells to them to form bodies of a higher, more complex, and gradually of a more special organization. This vitality may be called the Life of the Cell, resulting from chemical force.

Our cell, then, formed by a chemical law of affinity and assuming new and complex powers of a vital character, becomes a being, having an independent existence and capable of maintaining itself in a congenial habitat. This habitat it finds in the primitive seas that bathed the early quiescent, silurian beach. This cell, thus endowed with a vital force which we call life, becomes a neucleus, around which cluster other cells to produce those first simple forms of life, beginning with the few celled protozoan and continuing on through the long line of animated beings up to man.

If our theory is correct, that the cell acquired its vitality or life through chemical force, the first step is made plain, and we can now go on and follow the law of evolution from the first dawn of life upon the earth through all its manifold and successive changes, tracing step by step living forms as they proceed to a higher and still higher form until we reach that level upon which we ourselves stand in the full enjoyment of a completely developed organization animated and controlled by a perfect brain. Assuming that cell-life began in the way we have indicated, how does it multiply and grow to produce others like itself. Fig. 1 shows the process of fission and growth of a cell to form others.

Fig. 1

At *a*, we have a single cell. *b*, cell dividing within its cell-wall into two. *c*, cell dividing into four. *d*, cell dividing into eight. *e*, cell finely divided into minute cells. *f*, rupture of cell wall and escape of young cells.

The young cells having escaped from the parent cell are ready to enter upon a new existence and they at once arrange themselves in a straight line to form a simple foot-stalk, with one end fastened to a rock as in Fig. 2.

Fig 2

*Row of cells attached to a rock in the sea.*

Upon the top of this simple line of cells others add themselves and we have the lowest form of sponge. A few Cilia or hair-like processes are developed upon the inner side to waft towards them the elements contained in the water which they use as food, as in Fig. 3.

Fig .3

*Cells uniting to form Sponge.*

The line of cells can be easily developed into higher forms of sponges, algæ, and crinoids, or it can detach itself to form a simple worm with a free movement in the water, enabling it to travel about in search of its food. This movement places it a step higher in the scale above its stationery fellow animals the sponges.

Fig 4

*Worm swimming about in search of Food.*

By the addition of cells and a higher development a low form of fish is evolved.

Fig .5

*Early form of simple fishes.*

Fig. 5 shows the earliest forms of these fishes and it will be noticed that the upper fin of the tail is larger than the lower. This form belongs to all the early fishes. It will be impossible to mention here any but a few of the most typical forms of life. Between the single cell and the sponge there is a vast multitude of Protozoan forms, systemless animals which fill up the gap between, and also between the simple worm and the fish, which has a vertebral column, there is a great array of intermediate forms which fill up that gap. The evolution of animal life is orderly and slow, not abrupt.

From the lower forms of fishes come the higher, and the evolution of life keeps pace with the evolution of the conditions of the earth, when the air becomes fit for respiration and is purer, then we have after the fishes, the lowest forms of reptiles. A modification of the fish gives us the reptile.

Fig. 6

*Early form of Reptilian life.*

During the long reptilian age these creatures grew to an enormous size, and later became so modified in form that they insensibly shaded into quadrupeds. There are many animals that seem to form " connecting links " between these saurians and quadrupeds, among them may be mentioned the Ramphorynchus, that curious reptile with longer legs than the earlier lizards, diminished tail and singular pointed wing-like appendages that foreshadows the bird as well as the quadruped. A little higher in the scale comes the Iguanodon, and higher still the Megalosaurus. Following another branch of evolution of this animal tree we see how from the saurians, emerge the animals that later are destined to form the apes. The kangaroo is but a modified lizard.

Fig. 7

*Lizard tilted up on one end to form a Kangaroo.*

As saurians progressed in structure and rose higher in the scale of animal life we find them gradually assuming herbivorous habits, and this led them to feed upon the foliage of shrubs and trees. To do this it became necessary to raise their anterior limbs from the ground in order to reach the trees, and this act during long lapses of time at last modified their anatomical structure and converted a fore limb originally intended for progression, into a prehensile hand. The sloths belong to this class. The kangaroo is a grade higher than the sloths, and later come the lower monkeys, after them the higher apes and those anthropoid animals, the orang and gorilla.

Assuming this view of the law of evolution, and we believe it is the correct and logical and scientific view, we see how during the lapse of immense periods of time animal forms have slowly been evolved from the lower to the higher. From the simple cell came clusters of cells, then strings of cells, which shaped the sponges, the crinoids, the worms and the vast numbers of trilobites which were only the string of cells bent round thus:

Fig. 8

*Arrangement of cells to form Trilobites.*

and three rows of cells added to form the tri-lobed animal.

The great sub-kingdoms of Radiates and Articulates, are but higher modifications of the strings of cells, modifications which slowly arose and progressed by the great law of evolution.

The early fishes were quite simple, the higher more complex, and in another age we see these higher fishes modifying their fins into flippers and legs, and the saurians come upon the scene.

The prodigious development of these creatures has led geologists to assign to them a separate age, the age of reptiles. and in this great epoch they must have found the earth a congenial habitat, and the conditions of life easy—for ease of existence and abundant food make animal life prolific—for they multiplied until they actually swarmed in those primitive seas. The enormous tails of these great lizards were organs of propulsion through the water in which they lived, the flippers simply guided their direction, as do the fins of fishes, but as they rose in the scale the flippers became legs, and we find them crawling on the muddy and swampy land.

Later they came upon higher land, and by the law of *Use* their legs became the organs of locomotion, and by the law of *Disuse* their tails degenerated and we have quadrupeds.

The improved condition of nature in the Mammalian age, the universally warm climate, the luxuriant vegetation gave a great impetus to the evolution of animal life. The abundance of food made the struggle for existence less fierce, the law of natural selection and of Use and Disuse changed and modified the forms of animals life, now so prolific and and abundant.

We see reptilian forms diverging more and more. Structural changes are taking place, the most striking of which are an increased development of the humerus, the radius and ulna in the anterior limbs and the femur, tibia and fibula in the posterior. This increased growth is a direct result of the increase of the area of dry land, making it necessary for the reptiles to have some other means of locomotion than the fin-like flippers that served to guide them in the sea. So during long epochs these changes took place, influenced by the law of Use.

As they developed these locomotory limbs their tails were no longer necessary for propulsion and so as we advance in the animal scale we see the caudae slowly atrophying until when we reach the higher animals we find them composed of degenerate vertebræ, having little or at most a secondary use, that of switching insects from their bodies. Even in the prehensile-tailed apes who use this organ for grasping, its use is secondary, for these animals never trust to it to *wholly* sustain the weight of their bodies, they merely use it in conjunction with a hand or foot. In the higher Authropoid Apes and in man the cauda has reached the extreme of degeneration through *disuse*

and we find it atrophied to four small bones which consolidate in adult age, having no office to perform and which is called the coccyx.

The change from the pachydermatous, horny hides of reptiles, which is composed of agglutinated epidermis, to that of Armadillo's, Hippopotami, Boars, Tapir's and Elephants up to the later mammals, as the Tiger, the Hyena, the Deer and the other softer skinned animals covered with, first, bristles, then coarse hair, then fine hair, is due to dry land and a dry atmosphere. The close, thick fur of the Bear, Fox, etc., came later with a change of climate.

The Reptilian Age closed when these great lizards slowly changed to quadrupeds. But what became of those strange and monstrous forms, such as the Icthyosaurus, the Pleisosaurus, the Iguanodon, the Megalosaurus, the Ramphonynchus, and many more besides that swarmed in those early seas. Their extinction was inevitable, it followed just as harmoniously and as sequentially as their evolution. They lived in shallow seas and muddy swamps. During long lapses of time these seas were slowly replaced by the gradual up-lift of the land, and the muddy swamps originally salt were slowly closed in by these uplifts, rains turned them into fresh water lagoons and finally they became dry land covered with a luxuriant vegetation.

These causes combined with the ever upward law of evolution were sufficient to extinguish most of this great species. Those that have lived to our own time, such as the alligator and crocodile, have become fresh water species and inhabit warm latitudes as they did in primitive ages.

In the Mammalian ages we notice a vast number of animal forms with most of which we are familiar to-day. A few such as the Anoplotherium, and others of the horse tribe, the Mammoth, the Mastodon, the Megatherium, and the great Sloths have passed away, leaving behind them their history in their fossil remains. The cause of the change from the Pig to the Elephant is to be found in the law of progression and favorable conditions of life as among the more important.

When we carefully study the Vertebrates we shall find that many of the differences between them are more apparent than real.

In the order Felidæ we find the osteology of these animals strikingly uniform, the real differences being in *size*, which is only relative, and in the color and markings of the hair, and this last is largely influenced by climate and natural selection. Their structure anatomically is the same, as also are their habits.

The Sheep, Goat, Llama, Guanaco, Deer, Camel, Giraffe are, scientifically speaking, identical. To the eye they present differences in size, color and hair. The first has fine hair or wool, all the rest have hair closely alike in texture, but their skeletons, stomachs, and outward habits are essentially the same.

This is true also of the Canidæ, it is true of the Ursidæ or Bears, it is true of all the different orders of Vertiebrate mammals.

The real differences between them are slight, the outward differences are due to modes of life, of habits, and of climate.

The fishes present few scientific differences. Their osteology shows a remarkable uniformity of structure. Size here again is relative. The differences of color are due to the same causes, viz: external influences and habits of life.

This is true again of the Birds. The causes of change in their form and color are due to climate and surrounding influences.

Speaking generally, and yet correctly, we may begin back, say with the Lizards, and regard the different orders—the Proboscidæ, Ursidæ, Felidæ, Canidæ, Hyenidæ, Bovidæ, Cervidæ, Camelidæ, Simiidæ—as different branches of the same animal tree, having fundamental similarities, but superficial points of variation, and these varying points are due to the spread of species over the the earth, the impress of climatic conditions, natural selection, "the survival of the fittist," the extinction of weaker forms, the geological changes in the earth's surface and the influence of food.

Perhaps the most startling changes in animal forms have been those brought about by the influences of Domestication. This has been more especially observed in the Horse, Cow,

Pig, Dog and Fowl, because these species have been brought under the immediate influence and absolute control of Man.

Beginning with the horse we see a great variety of forms from the heavily boned and massively built work-horse capable only of slow motion, but of enormous strength, to the fleet, thoroughbred racer, whose slender and agile limbs seemed clothed with the terrific speed of the locomotive engine. These changes in the equine form are wholly due to selection by man to crossing and to interbreeding. A man having a mare with certain qualities which he desires to perpetuate and improve will select with the greatest care a stud having like or superior qualities and pair her with him. If he desired strength or speed he will cross his mare with this special object in view and the mare will transmit her good qualities, thus improved by crossing, to her foal.

Men who breed horses for the turf know that the quality of speed is transmitted and so they choose those having a long ancestral record to cross with and breed from. The breeding of thoroughbreds has been raised to a fine art, and breeders study with minute zeal the poise of the head, the width and depth of the neck, the shoulder, chest and leg, knowing that these "horsey points" have much to do with the excellence of the animal and consequently his price.

With the cow, the quality of the milk and the flesh have been objects of the greatest care. Some, like the Alderney, have been bred for their milk-giving power alone; others, and notably the Durham, have been bred solely for the quality of their flesh as food. Even in Texas and the west, where no special object has been had in view, cattle owners have tried to improve the general excellence of their stock.

Pigs come under the same law. They vary by the same influences of domestication regulated by Man. In these animals we see every variety of form from the lean, gaunt wild boar, with long legs and flattened sides, to the improved Suffolk, which is almost a shapeless mass of fat.

Perhaps we see in the Dog the most striking change induced by domestication.

From the dainty skye and the delicate-limbed black-and-tan to the ferocious bull-dog and the heavily-boned mastiff we see every variety of form and color.

The practice of breeding in and in, or of pairing a brother and sister, which is the closest relation of consanguinity, has a vast influence in continuing and increasing certain traits or qualities. Color, size, shape of head and limbs are affected by this practice, and a great degree of *fineness* and purity of blood is attained by it.

The ordinary black-and-tan has been so selected and crossed with the best specimens of his kind that elegant animals with finely shaped heads and legs have been produced.

The bloodhound has been bred to improve his sense of smell and his olfactory bulbs and nerve filaments have been actually increased in size.

Pointers show the effects of selection and close breeding in a high degree.

All these varieties of the Canidæ which have sprung from a common stock have been produced by a long continued selection, close interbreeding and crossing. The changes in form have been accomplished with comparative ease by the absolute dominion of Man over these animals.

With Fowls precisely the same thing is true. The different strains of varying purity are the result of selection crossing and interbreeding under a long domestication.

With Birds in the natural state, the varieties seen in them are due to climate, habits and food through considerable lapses of time.

If this same law of selection could be applied to Man, if in marriage men and women could be selected and crossed with the especial view of transmitting certain good qualities and of suppressing certain bad qualities if men who are healthy, sane, and temperate could marry women who are also healthy, sane, and temperate, we should see not only an improvement in the physique of their offspring, but a positive decrease in the sum of insanity, intemperance and misery upon the earth.

## Figures and Tables

**Fig. 1 f1:**
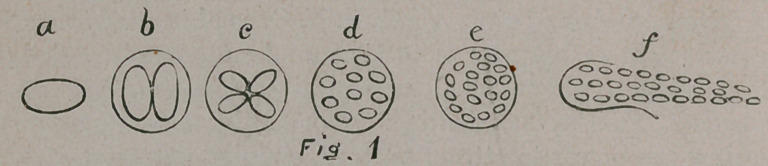


**Fig 2 f2:**
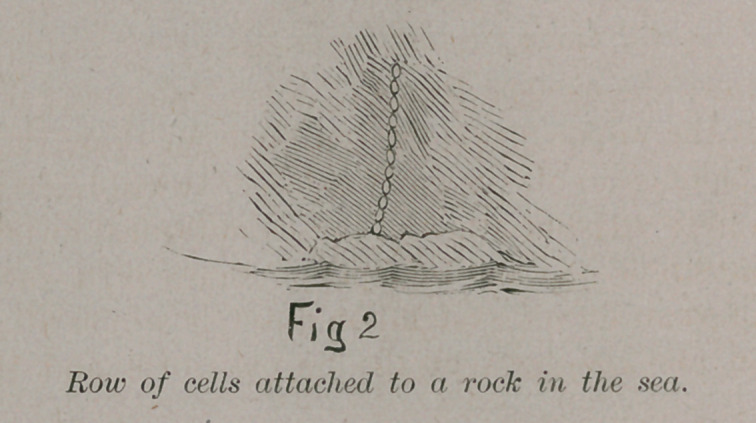


**Fig. 3 f3:**
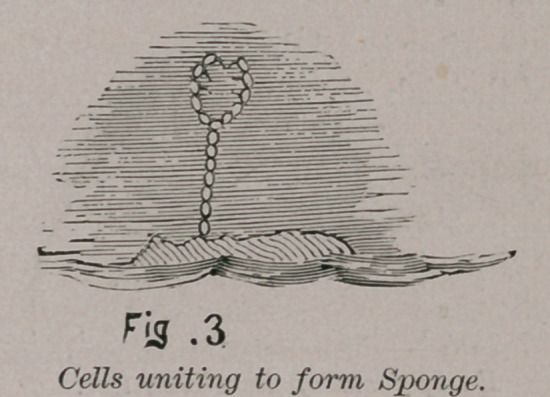


**Fig 4 f4:**
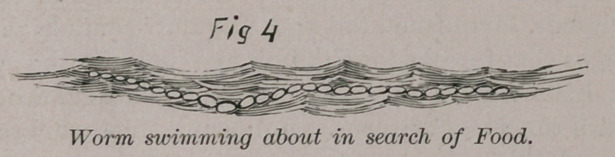


**Fig. 5 f5:**
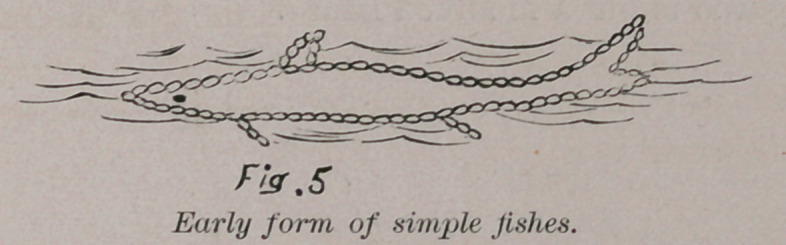


**Fig. 6 f6:**
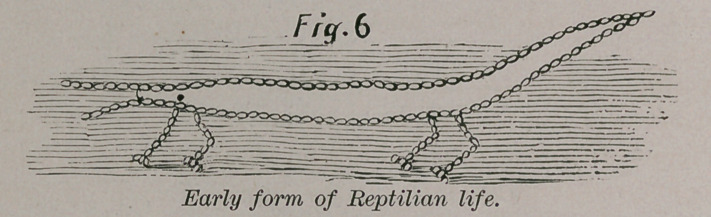


**Fig. 7 f7:**
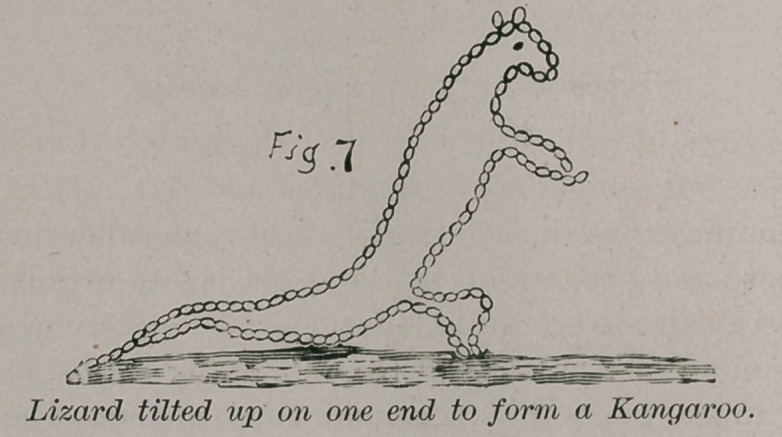


**Fig. 8 f8:**